# E-cigarette use in Brazil—prevalence and associated factors in a national cross-sectional study of 16,093 adults: a short communication

**DOI:** 10.1590/1516-3180.2025.3562.23022026

**Published:** 2026-05-18

**Authors:** Hannae Coelho Damasceno-de-Freitas, André Pontes-Silva, Renata Nogueira Duran Marquez-de-Souza, Francisco Winter dos Santos Figueiredo, Fernando Rodrigues Peixoto Quaresma, Erika da Silva Maciel

**Affiliations:** I Program in Teaching in Science and Health, Universidade Federal do Tocantins, Palmas (TO), Brazil.; II Postgraduate Program in Physical Education, Universidade Federal do Maranhão, São Luís (MA), Brazil.; III Program in Teaching in Science and Health, Universidade Federal do Tocantins, Palmas (TO), Brazil.; IV Program in Teaching in Science and Health, Universidade Federal do Tocantins, Palmas (TO), Brazil.; V Program in Teaching in Science and Health, Universidade Federal do Tocantins, Palmas (TO), Brazil.; VI Program in Teaching in Science and Health, Universidade Federal do Tocantins, Palmas (TO), Brazil.

**Keywords:** Nicotine, Public health, Epidemiologic surveillance services, Electronic cigarette use, Prevalence, Brazil

## Abstract

**BACKGROUND:**

Monitoring tobacco consumption is important for supporting national and global agendas and commitments.

**OBJECTIVE:**

To analyze the prevalence and sociodemographic and geographic factors associated with e-cigarette use in Brazil, using data from Vigitel 2023.

**DESIGN AND SETTING:**

Cross-sectional analytical study was conducted using secondary data from Vigitel 2023.

**METHODS:**

Sociodemographic characteristics (sex, age, education, and ethnicity), geographic location, and e-cigarette use were analyzed. Descriptive analyses were performed using Chi-squared and Mann–Whitney ***U***-tests. Additionally, weighted multivariable logistic regression models were used to examine independent associations between sociodemographic factors and current e-cigarette use. Post-stratification weights were applied, and adjusted odds ratios (aOR) with 95% confidence intervals (95% CI) were estimated.

**RESULTS:**

Among 16,093 adults, the prevalence of current e-cigarette use was 5.55%. In the adjusted model, younger age was strongly associated with use (aOR per year increase = 0.93; 95% CI 0.92–0.94; *p* < 1). Men had higher odds of use than women (aOR = 1.34; 95% CI 1.12–1.6; *p* < 0.01). Educational level was not independently associated with usage. Caucasian individuals had higher odds compared to mixed-race individuals (aOR = 1.29; 95% CI 1.05–1.58; ***p*** = 0.015). Residents of the southern region showed increased odds (aOR = 1.48; 95% CI 1.1–1.99).

**CONCLUSION:**

Current e-cigarette use in Brazil is independently associated with younger age, male sex, Caucasian ethnicity, and residence in southern Brazil. These findings underscore the importance of strengthening public policies that regulate and control these devices as well as educational campaigns targeting high-risk groups.

## INTRODUCTION

 Smoking is responsible for approximately eight million deaths annually. Of these deaths, approximately seven million are due to direct tobacco consumption and approximately 1.2 million are a consequence of exposure to secondhand tobacco smoke. Therefore, smoking is considered a serious global public health problem and a risk factor for diseases such as lung cancer, chronic obstructive pulmonary disease, myocardial infarction, stroke, type 2 diabetes, hypertension, infertility, oral health issues, and premature skin aging.^1^

 Nicotine, the main psychoactive substance in cigarettes, strongly contributes to respiratory diseases such as chronic obstructive pulmonary disease and asthma, and cardiovascular problems such as atherosclerosis and stroke. Nicotine also affects the digestive system, causing gastroesophageal reflux, peptic ulcers, and liver cirrhosis. It also affects the genitourinary system, causing erectile dysfunction and infertility.^2^

 E-cigarettes, also known as electronic smoking devices, have many names including vapors, hookah pens, e-hookahs, e-cigars, personal vaporizers, and mods. Although these drugs have begun to act as alternatives to smoking cessation, they pose significant public health risks. The incorrect perception that e-cigarettes are harmless has contributed to their popularity, particularly among young people.^3,4^

 The use of e-cigarettes exposes the body to chemicals such as nicotine, formaldehyde, acetaldehyde, acrolein, and diacetyl, as well as heavy metals such as nickel, cadmium, and chromium. These compounds include carcinogens and cytotoxic substances that cause lung and cardiovascular diseases. Furthermore, electronic cigarette use is concerning because it can lead to the consumption of other tobacco products, particularly among adolescents and young adults.^5^

 A study conducted by the Heart Institute (involving 400 e-cigarette users in São Paulo, Brazil) found that their nicotine levels were up to six times higher than those of conventional cigarette smokers who smoked an average of 20 cigarettes per day. This suggests that e-cigarettes pose a greater risk of addiction. 

 Additionally, the Covitel report, which surveyed 9,000 people via telephone about the risk factors for chronic noncommunicable diseases during the pandemic, indicated that the prevalence of e-cigarette use in Brazil was 8% in the first quarter of 2023, primarily among those aged 18–24.^1^

 Although national surveys such as the Brazilian National School Health Survey (PeNSE) and the Covitel study have reported the prevalence of e-cigarette experimentation and use in specific populations, particularly adolescents and young adults, there is still limited evidence based on nationally standardized surveillance systems that allow comparisons across all Brazilian state capitals. Moreover, most Brazilian studies have focused on adolescents, university students, or convenience samples, and few have explored the sociodemographic and geographic distribution of e-cigarette use in the adult population using population-based data.^3,4^

 Internationally, surveillance systems, such as the Behavioral Risk Factor Surveillance System in the United States and similar national health surveys in Europe, have provided consistent monitoring of e-cigarette use trends and associated factors. However, comparable analyses of chronic disease risk factors using Brazil’s official surveillance system are scarce.^6^

 Therefore, analyzing Vigitel 2023 data provides an opportunity to fill this gap by offering updated, population-based estimates of e-cigarette use among adults living in all Brazilian state capitals and identifying associated sociodemographic and regional factors. This information is essential to guide regulatory policies and targeted public health interventions.^7^

## OBJECTIVE

 Therefore, given the limited adult population-based evidence derived from Brazil’s official surveillance systems and the need for updated nationwide data, this study sought to analyze the prevalence and sociodemographic and geographic factors associated with e-cigarette use in Brazil, using data from Vigitel 2023. 

## METHODS

 This was a cross-sectional analytical study using secondary data from the Surveillance of Risk and Protective Factors for Chronic Diseases by Telephone Survey (Vigitel) 2023.^8^ Vigitel is an annual, population-based survey conducted by the Brazilian Ministry of Health through telephone interviews with adults (aged 18 and older) living in the capitals of Brazil’s 26 states and the Federal District. This study was conducted in accordance with the principles of the Declaration of Helsinki. The data used in this study are publicly available, and it was not possible to access identifying information concerning the participants. Furthermore, this study adhered to Resolution No. 510 of the 2016 National Health Council (Brazil).^8^

 The 2023 Vigitel sample consisted of 16,093 respondents with 800 interviews conducted in each capital city, equally divided between landlines and cell phones. Vigitel’s methodology employs statistical weighting to adjust the sample distribution to the adult population living in households with telephone access, using data from the Brazilian Institute of Geography and Statistics’ Demographic Census.^8^ The data used in this study are publicly available, and it is not possible to access identifying information about the participants. Furthermore, this study adhered to Resolution Number 510 of the 2016 National Health Council (Brazil).^8^

 A total of 16,093 respondents were included in the analysis. Current e-cigarette use was defined as daily or occasional use of electronic nicotine delivery devices. 

 The independent sociodemographic variables included sex (male/female), age (years), education level ( high school, college, or postgraduate), and ethnicity/color (Caucasian, black, mixed, or other). 

 The city of residence (26 state capitals and the Federal District) was used for descriptive analyses. For multivariate analyses, the city of residence was grouped into five macro-regions (North, Northeast, Southeast, South, and Central-West) to improve model stability and interpretability. 

 Descriptive analyses were conducted to estimate the prevalence and compare groups using the Chi-squared and Mann–Whitney *U*-tests. Subsequently, weighted multivariable logistic regression models were used to assess the independent associations between sociodemographic variables and current e-cigarette use. Post-stratification (raking) weights were applied to account for the Vigitel sampling design. Adjusted odds ratios (aOR) and 95% confidence intervals (95% CI) were calculated. All variables were included in the final model. Statistical significance was set at *p* < 0.05. 

## RESULTS

 Among the 16,093 adults interviewed in 2023, 5.55% reported current e-cigarette usage. In the descriptive analyses, significant differences were observed according to sex, age, ethnicity/color, and city of residence (Tables 1 and 2). In the weighted multivariable logistic regression model (Table 3), younger age remained strongly associated with e-cigarette use (aOR per year increase = 0.93; 95% CI 0.92–0.94; *p* < 1). Men had 34% higher odds of use than women ((aOR = 1.34; 95% CI 1.12–1.6; *p* < 0.01). Caucasian individuals showed higher odds compared to mixed-ethnicity individuals (aOR = 1.29; 95% CI 1.05–1.58; *p* = 0.015). Regionally, residents of the South had higher odds of use (aOR = 1.48; 95% CI 1.1–1.99), while residents of the northeast showed lower odds compared to the north region (aOR = 0.74; 95% CI 0.55–0.98). 

**Table 1:** Factors associated with the use of electronic cigarettes (n = 16,093)

**Variables**		**Electronic Cigarette**			
		**No**	**Yes**	**Total**	Test*
		**15,200 (94.45%)**	**893 (5.55%)**	**16,093 (100%)**	
Sex					< 1
	*Male*	5,806 (38.2%)	510 (57.11%)	6,316 (39.25%)	
	*Female*	9,394 (61.8%)	383 (42.89%)	9,777 (60.75%)	
	Age (mean ± SD)	47.07 (± 16.85)	31.37 (± 11.94)	46.20 (± 17)	< 1
Education Level					0.12
	*High school*	7,601 (50.01%)	443 (49.61%)	8,044 (49.98%)	
	*College degree*	5,773 (37.98%)	361 (40.43%)	6,134 (38.12%)	
	*Graduate degree*	1,826 (12.01%)	89 (9.97%)	1,915 (11.9%)	
Race/Color					< 1
	*White*	5,892 (38.76%)	408 (45.69%)	6,300 (39.15%)	
	*Black*	1,611 (10.6%)	82 (9.18%)	1,693 (10.52%)	
	*Asian*	175 (1.15%)	13 (1.46%)	188 (1.17%)	
	*Mixed-race (pardo)*	7,008 (46.11%)	363 (40.65%)	7,371 (45.8%)	
	*Indigenous*	142 (0.93%)	10 (1.12%)	152 (0.94%)	
	*Other*	254 (1.67%)	9 (1.01%)	263 (1.63%)	
	*Doesn’t know*	77 (0.51%)	7 (0.78%)	84 (0.52%)	
	*Prefer not to answer*	41 (0.27%)	1 (0.11%)	42 (0.26%)	

* Mann–Whitney *U*-test was used for quantitative variables (age and education level) or Chi-squared test was used for categorical variables (sex, ethnicity, and city).

**Table 2:** Factors associated with electronic cigarette use in Brazil’s regions (n = 16,093)

**Variables**		**Electronic Cigarette**			
		**No**	**Yes**	**Total**	Test*
		**15,200 (94.45%)**	**893 (5.55%)**	**16,093 (100%)**	
City					< 1
	*Aracaju*	581 (3.82%)	11 (1.23%)	592 (3.68%)	
	*Belém*	582 (3.83%)	23 (2.58%)	605 (3.76%)	
	*Belo Horizonte*	537 (3.53%)	28 (3.14%)	565 (3.51%)	
	*Boa Vista*	597 (3.93%)	41 (4.59%)	638 (3.96%)	
	*Campo Grande*	478 (3.14%)	47 (5.26%)	525 (3.26%)	
	*Cuiabá*	557 (3.66%)	42 (4.7%)	599 (3.72%)	
	*Curitiba*	515 (3.39%)	63 (7.05%)	578 (3.59%)	
	*Florianópolis*	549 (3.61%)	62 (6.94%)	611 (3.8%)	
	*Fortaleza*	545 (3.59%)	21 (2.35%)	566 (3.52%)	
	*Goiânia*	529 (3.48%)	44 (4.93%)	573 (3.56%)	
	*João Pessoa*	599 (3.94%)	13 (1.46%)	612 (3.8%)	
	*Macapá*	631 (4.15%)	15 (1.68%)	646 (4.01%)	
	*Maceió*	563 (3.7%)	27 (3.02%)	590 (3.67%)	
	*Manaus*	618 (4.07%)	19 (2.13%)	637 (3.96%)	
	*Natal*	556 (3.66%)	36 (4.03%)	592 (3.68%)	
	*Palmas*	616 (4.05%)	35 (3.92%)	651 (4.05%)	
	*Porto Alegre*	535 (3.52%)	36 (4.03%)	571 (3.55%)	
	*Porto Velho*	542 (3.57%)	32 (3.58%)	574 (3.57%)	
	*Recife*	575 (3.78%)	18 (2.02%)	593 (3.68%)	
	*Rio Branco*	532 (3.5%)	31 (3.47%)	563 (3.5%)	
	*Rio de Janeiro*	576 (3.79%)	30 (3.36%)	606 (3.77%)	
	*Salvador*	590 (3.88%)	16 (1.79%)	606 (3.77%)	
	*São Luís*	599 (3.94%)	27 (3.02%)	626 (3.89%)	
	*São Paulo*	487 (3.2%)	59 (6.61%)	546 (3.39%)	
	*Teresina*	560 (3.68%)	32 (3.58%)	592 (3.68%)	
	*Vitória*	589 (3.88%)	26 (2.91%)	615 (3.82%)	
	*Brasília*	562 (3.7%)	59 (6.61%)	621 (3.86%)	

* Mann–Whitney *U*-test was used for quantitative variables (age and education level) or Chi-squared test was used for categorical variables (sex, ethnicity, and city).

**Table 3:** Weighted multivariable logistic regression analysis of current e-cigarette use, Vigitel 2023 (n = 16,093)

**Variable**		**Adjusted OR**	**95% CI**	***p* value**
Age (per year increase)		0.93	0.92–0.94	< 0.01
Sex				
	*Male (vs female)*	1.34	1.12–1.6	< 0.01
Education Level				
	*College (vs ≤ high school)*	1.18	0.97–1.44	0.09
	*Postgraduate (vs ≤ high school)*	0.88	0.62–1.24	0.46
Race/Color				
	*White (vs mixed-race)*	1.29	1.05–1.58	0.015
	*Black (vs mixed-race)*	0.91	0.66–1.26	0.58
	*Other (vs mixed-race)*	1.12	0.73–1.72	0.6
Region				
	*Northeast (vs North)*	0.74	0.55–0.98	0.038
	*Southeast (vs North)*	1.21	0.93–1.57	0.15
	*South (vs North)*	1.48	1.10–1.99	0.01
	*Center-West (vs North)*	1.33	0.99–1.79	0.056

Model adjusted for age, sex, education level, ethnicity, and macroregion. Post-stratification weights (pesorakes) were applied. Reference categories: female; ≤ high school; mixed-ethnicity; North region. OR, odds ratio; CI, confidence interval.

## DISCUSSION

 The findings of this study indicate that while e-cigarette use in Brazil is still restricted to a portion of the population, it is primarily concentrated among young people with a significantly lower average age than non-users. These results expand on the previous Brazilian evidence by providing updated estimates based on a nationally standardized surveillance system covering all state capitals, thereby complementing the findings of school-based and pandemic-period surveys. 

 The inverse association between age and e-cigarette use indicated that younger adults were disproportionately affected. This highlights the emergence of a new generation exposed to nicotine without prior experience with conventional smoking, which makes this group particularly vulnerable to addiction.^9-11^

 After adjusting for potential confounders, younger age remained the strongest predictor of e-cigarette use. Male sex and Caucasian ethnicity were independently associated with higher odds of use, whereas educational level was not significantly associated after adjustment. Regional disparities persist, particularly in the South region.^12,13^

 In terms of geographic distribution, e-cigarette use was most prevalent in Curitiba, São Paulo, and the Federal District, with statistically significant differences observed among these locations. These findings support the notion that large cities, with their economic and social dynamics, can act as hubs for disseminating new nicotine products and influencing consumption behavior.^9^

 Despite the 2009 ban imposed by the National Health Surveillance Agency (Agência Nacional de Vigilância Sanitária), reinforced in 2024, e-cigarette use continues to grow among young people, indicating that current strategies are insufficient to curb this trend. Furthermore, the smuggling and illegal marketing of these products poses additional challenges, requiring increased oversight and strengthened public control policies.^8^

 E-cigarette use in Brazil raises significant public health concerns, particularly given the false perception that these devices are less harmful than conventional cigarettes. Although e-cigarettes eliminate tobacco combustion, they still expose users to high concentrations of nicotine, heavy metals, and toxic compounds in aerosols generated by vaporization.^14^

 Furthermore, attractive flavors and modern designs contribute to the popularity of these products among young people, creating an environment that encourages the early initiation and development of nicotine dependence. Evidence also shows that e-cigarette use can lead to conventional smoking. Thus, the use of these devices not only challenges the healthcare system, but also threatens the progress of historic anti-smoking policy in Brazil.^10,11^

 Another important factor is the economic impact of smoking, which extends beyond health issues. The WHO warns that tobacco product use contributes to poverty by diverting essential financial resources such as those allocated to food and housing. While e-cigarette use appears to be associated with younger, Caucasian, and more educated men, conventional cigarette use has different characteristics. 

 The National Health Survey revealed that approximately 10% of Brazilians spend money on tobacco products and tend to have lower income and education levels than non-smokers. The National Cancer Institute indicates that cigarette smoking accounts for approximately 8% of per capita household income in Brazil. This finding highlights the relationship between tobacco spending and the limited resources required for other necessities. This contributes to a cycle of socioeconomic vulnerability that exacerbates public health problems and limits access to adequate care.^13^

 Therefore, to improve the effectiveness of existing public policies, it is important to recognize that strategies for reducing the use of both types of cigarettes must differ. Brazil has adopted measures in response to this scenario, such as the Strategic Action Plan to Combat Chronic Noncommunicable Diseases (2011–2022). This plan aims to reduce smoking prevalence by 30%. However, as this target was not fully achieved, the commitment was renewed in the plan to combat noncommunicable diseases and injuries (2021–2030), which was launched in 2022. Smoking reduction is a central objective of this plan. The plan emphasizes the necessity of stronger public policies, particularly considering the increased use of e-cigarettes, which are still considered a "less harmful" alternative to traditional cigarettes.^12^

 Despite the National Health Surveillance Agency’s bans since 2009, the use of electronic smoking devices continues to increase, suggesting that the current monitoring and control strategies are ineffective. Furthermore, the use of flavorings in cigarettes and the smuggling of these products are critical issues requiring a more intensive approach.^13^

 Therefore, it is essential to redirect public policies toward e-cigarettes and intensify educational initiatives focused on health literacy for self-care, especially in regions and groups where e-cigarette use is most prevalent. Therefore, strengthening educational campaigns with an emphasis on demystifying the benefits of e-cigarettes is crucial for preventing the growth of e-cigarette use among young people and reducing the social and economic impact of smoking in Brazil.^13^

 The results of Vigitel 2023 provide a solid foundation for understanding the profile of e-cigarette users in Brazil and can contribute to strengthening public policy. Continuous monitoring of factors associated with the use of these substances and implementation of educational interventions are essential to address the challenges posed by chronic, noncommunicable diseases in Brazil.^9,14^

 In summary, the growth in e-cigarette use in Brazil, especially among young people, highlights the urgent need to strengthen health education initiatives as a central axis of prevention strategies. Investing in qualified, accessible, and ongoing information is essential to curb this trend and protect new generations from the risks associated with early nicotine exposure and the development of addiction. 

## CONCLUSION

 In Brazil, current e-cigarette use was independently associated with younger age, male sex, Caucasian ethnicity, and residence in the southern region. These findings highlight the need for targeted public health policies and continuous surveillance using population-based monitoring systems. Therefore, it is imperative to strengthen public policies that limit access to e-cigarettes and expand educational initiatives to inform people concerning the associated health risks. In addition, monitoring the prevalence and determinants of e-cigarette use should be a priority in public health agendas to support preventive measures aligned with national and international tobacco control goals. 

## Data Availability

Data supporting the findings of this study are available upon request from the corresponding author, André Pontes-Silva.
